# Use of Microbes for Improving Food Safety and Quality

**DOI:** 10.1155/2018/3902698

**Published:** 2018-09-25

**Authors:** Nevijo Zdolec, José Manuel Lorenzo, Ramesh Chandra Ray

**Affiliations:** ^1^Department of Hygiene, Technology and Food Safety, Faculty of Veterinary Medicine, University of Zagreb, Heinzelova 55, 10000 Zagreb, Croatia; ^2^Centro Tecnológico de la Carne de Galicia, Rua Galicia No. 4, Parque Tecnológico de Galicia, San Cibrao das Viñas, 32900 Ourense, Spain; ^3^Centre for Food Biology Studies, 1071/17 Jagamohan Nagar, Khandagiri PO, Bhubaneswar 751 030, Odisha, India

Food safety and quality depend on many specific factors, including favorable or harmful microbial properties. The concept of food safety changed in recent years from hazard-based to risk-based approach and preventive interventions are needed from preharvest level to food processing. Use of microbes in primary production may be beneficial for health and productivity of food-producing animals, as well as quality and safety of animal products intended for human consumption. Antimicrobial capacities of selected microbes should be employed in developing of new food preservation methods and food spoilage prevention. Many foodborne threats to consumers' health could also occur during food processing and storage, and many competitive microbes can be used to prevent or reduce the risk.

Hence, the power of microbes as a tool of upgrading food safety and quality may be used through the whole agrofood chain. Some recent topics in this scientific area are reduction of foodborne hazards by preharvest interventions, improvement of food quality by microbe-based feed modifications, prevention of food spoilage and extending shelf-life by microbial interactions, probiotics in feed and food, autochthonous and commercial starter cultures' properties, and reduction of foodborne public-health hazards (i.e., pathogens, spoilers, biogenic amine producers, mycotoxins producers, and carriers of antimicrobial resistance) by applying competitive microbes or their metabolites in food.

This approach was followed by selected papers within the special issue, including topics from soil to cheese and meat. Study of L. T.-H. Tan et al. explored the antioxidant potential of* Streptomyces *sp. from mangrove sediments since it is believed that stress-exposed mangrove-related microbes develop specific antioxidant metabolites which could have a high practical importance in food processing, i.e., prevention of food lipid oxidation. The strain MUM292 showed a strong antioxidant potential by producing bioactive compounds with radical scavenging and metal-chelating activities. This study showed wide opportunities of natural microbe-delivered bioactive compounds from exotic niches (mangrove soil) in improving food safety and quality. The second study, presented by J. Mei et al., emphasizes the importance of chemical treatments in plant production and evident impact on food safety along the whole food chain. In this respect, authors investigated the fungal strain* Trichoderma harzianum* Th33 as biocontrol agent with important role in disease prevention, improving the quality of agricultural products and reduction of pesticide hazards. Copper-tolerant* Trichoderma* can be used in combination with copper-containing pesticides and therefore reduce the use of chemical pesticides. In addition, it can also be used for bioremediation of copper-contaminated soils since copper pollution is disadvantageous to the quality and safety of food. Authors showed that C2H2 transcription factor gene* thmea1* negatively regulates the copper tolerance of* Trichoderma harzianum* Th33. Studying the function of* thmea1* will help reveal the copper tolerance mechanism of* Trichoderma*. The study of S. Marcinčák et al. involved the approach of improving quality properties of poultry meat by modification of their diet using fungal fermentation compounds. The fermented bioproduct was prepared by fermentation of corn meal by filamentous fungi* Umbelopsis isabellina* CCF 2412 in solid-state fermentation process and final bioproduct was enriched with gamma-linolenic acid and *β*-carotene. Application of fermented bioproduct into commercial feed mixture positively influenced profile of fatty acids in breast meat. The amount of gamma-linolenic, alpha-linolenic, and oleic acids in fat of breast muscles was increased and n-6/n-3 ratio has been also changed. Oxidative stability of fat and sensory properties of the meat during the storage (4°C, 7 days) of meat were not affected by fermented bioproduct. Authors emphasized that solid-state fermentation in feed production is an interesting form of production of significant fatty acids and beta-carotene to enrich the poultry diet with these important ingredients, which subsequently increases the share of important PUFAs and the oxidative stability of fats in the produced meat. Filamentous fungi were also in focus in the study performed by S. Cosentino et al., but as unfavorable microbes involved in cheese spoilage. The authors investigated antifungal activities of lactobacilli* in vitro *and applied the selected potential antifungal cultures in cheese simultaneously inoculated with spoilage species of* Penicillium *and* Aspergillus. The *authors showed that selected lactobacilli reduced the fungal population in both contaminated milk and cheese, as well as delaying the mycelial growth on the cheese surface. Selected antifungal cultures did not show any negative effect on standard starter culture applied or sensorial properties of cheese and can be considered as protective cultures in cheese making. Finally, S. S. Behera et al. reviewed functional properties of* Lactobacillus plantarum *for improving safety properties of fermented foods. Lactic acid bacteria in general are known as beneficial microbes in fermented foods and may contribute to human health (probiotics). The properties of* Lactobacillus plantarum *are discussed within the safety and quality aspects in traditional food systems, biotechnology (enzyme systems, bioactive metabolites, vitamins, and sensorial attributes), and health considerations (bacteriocins, probiotics, antioxidant, antibacterial, and antifungal activities). This review emphasizes that the* Lb. plantarum* strains with their probiotic properties can be of great benefit against harmful microflora (foodborne pathogens) to increase safety and shelf-life of fermented foods.

As guest editors, we hope that the presented special issue will fulfill the expectations of readers and will contribute to developments in safety and quality of food by power of microbes.

